# Nurses' Perspectives on Inpatient Care Responsiveness at the Gazan Public Hospitals

**DOI:** 10.4314/ejhs.v31i4.20

**Published:** 2021-07

**Authors:** Iyad Ibrahim Shaqura, Ebrahim Jaafaripooyan, Mostafa Hosseini, Abed El Raheem Shaban Shagora, Ali Akbari Sari

**Affiliations:** 1 Department of Health Management and Economics, School of Public Health, Tehran University of Medical Sciences, Tehran, Iran; 2 Department of Health Management and Economics, School of Public Health, Tehran University of Medical Sciences, Tehran, Iran; 3 Department of Epidemiology and Biostatistics, School of Public Health, Tehran University of Medical Sciences, Tehran, Iran; 4 Ministry of Health, Gaza Governorates, Palestine; 5 Department of Health Management and Economics, School of Public Health, Tehran University of Medical Sciences, Tehran, Iran

**Keywords:** Responsiveness, Hospitals, Inpatient care, Nurses, Gaza, Palestine

## Abstract

**Background:**

Responsiveness is one of the intrinsic goals of health systems. This study aimed at assessing the responsiveness of inpatient care in accordance to nurses' perspectives, particularly in internal medicine ‘medical’ and surgical departments, at the Gazan public general hospitals in 2020.

**Methods:**

This cross-sectional descriptive study was conducted at 5 public general hospitals in Gaza. Data were collected from 277 nurses using an interview-based questionnaire composed mainly of 36 items to measure responsiveness on a 4-point Likert scale. Descriptive statistics, independent t-test and analysis of variance (ANOVA) using SPSS 22.0.

**Results:**

The overall responsiveness was about 77.5%. Access to social support was the highest-performing domain but it was the less important. Dignity was the second-highest in performance but the most important domain. Choice of provider and quality of basic amenities were almost the lowest in both performance and importance. Hospital, marital status, educational level, position at work, income, department, and the experience in the current ward have led to significant differences in the level of responsiveness.

**Conclusion:**

Supply-side should be considered to delineate the status quo of responsiveness accurately. There is a room for further improvement in the interpersonal domains of responsiveness without extravagant expenditures. Policymakers need to emphasize on better allocation of budget for client-orientation domains of responsiveness as well. Hospital characteristics had a pivotal role in creating significant differences among respondents. Likewise, socioeconomic status and cultural diversity of nurses led to significant variations in their responses, hence, this calls for robust and well-designed researches, including non-public hospitals, to determine the most influential factors.

## Introduction

Besides health status and financial fairness, responsiveness has been recognized by the World Health Organization (WHO) as the third intrinsic goal of any health system. In addition, it can be deemed as a tool for measuring the health system performance (1).

In the recent years, the importance of responsiveness as a performance tool was an area of interest in different conferences and reinforced by various institutes such as the European Ministerial Conference on Health Systems (2) and the National Institute for Health and Care Excellence (3).

Responsiveness can be defined as “the capacity of health system to satisfy the non-clinical expectations of people, in addition to the way by which they are treated, and the environment in which health services are provided to them” (4). Hence, responsiveness aims at raising the level of patients' satisfaction through responding to their needs regardless of responsiveness impact on the health status (5,6). Furthermore, improvement of health system responsiveness is not costly, in terms of finance, equipment and workforce, if compared with the clinical aspects of healthcare (7).

Responsiveness is viewed via two aspects; the level ‘*goodness*’ and the distribution ‘*fairness*’. The level of responsiveness can be measured through the performance of domains, and ultimately the overall responsiveness, while, the distribution shows the extent to which services are fairly provided to individuals in the society (8). Responsiveness of inpatient care comprises eight domains which are classified into two categories: 1) interpersonal set which embraces dignity (DIG), clear communication (CC), autonomy (AUT) and confidentiality (CON); and 2) client orientation set which encompasses prompt attention (PA), access to social support networks (ASN), choice of provider (CP) and quality of basic amenities (QBA) (8,9).

In the literature, studies on responsiveness have mostly emphasized on the demand side by assessing the correlation between sociodemographic characteristics of patients and the reported level of responsiveness. Nevertheless, little is yet known about the influence of supply-side factors, therefore, few studies have investigated the supply-side determinants of responsiveness with countries taken as units of observation (10). Stressing on supply-side factors, such as hospital-specialty, medical departments and wards, and health-care providers, is substantial in a comprehensive evaluation of responsiveness (12).

Previous studies demonstrated some differences in the perceptions of nurses and patients regarding caring relationship (13). It is noteworthy that more exposure to nursing care leads to improve patients' perception about the health-care services they receive (14). In one study, it was found that nursing staff workload had a strongly negative association with patients' responsiveness on CC and CON domains; meanwhile, this relationship was not significant in the case of physicians' workload. Moreover, the expenditure on nursing staff had positive and strong impact on the level of all domains. Furthermore, educating and training health staff, including nurses, was remarkably correlated to raising the levels of all responsiveness domains. The study also revealed a negative relationship between the number of hospital beds and the performance of all responsiveness domains, conversely, patients case mix (i.e. different groups of patients and types of their diagnosis) has positively influenced on the level of domains (10).

In Javadi et al study, they compared between nurses and patients based on their perspectives on the responsiveness of Iranian hospitals. They found a difference between nurses and patients regarding the level of responsiveness, but this was not statistically significant. Moreover, the differences among nurses according to their sex, educational level and type of workplace were not also significant (15).

**Study context**: The Ministry of Health (MOH) is the main provider among the five providers in the Palestinian health system, which owns and administers 471 primary health-care centers and 26 hospitals in both the West Bank and Gaza Strip (GS). There are 13 MOH hospitals in the GS containing about 2,343 active beds (11.6 bed per 10,000 inhabitants). Of them, 7 general hospitals provide medical and surgical inpatient services, 5 mainly and 2 partially. These hospitals are distributed over the five governorates in the GS. Active beds in the medical and surgical departments stand for 52.7% of the total installed beds at the public hospitals in the GS (16).

As the first in the Palestinian health sector, this study aimed at assessing the responsiveness of inpatient care based on nurses' perspectives, particularly in medical and surgical departments, at the Gazan public general hospitals in 2020.

## Methods and Materials

**Study setting**: This cross-sectional descriptive study was undertaken in the medical and surgical departments which cover a wide range of inpatient services at the Gazan public general hospitals in 2020. It should be noted that no intervention was directed to improve the knowledge nor raise the awareness of nurses about responsiveness prior to/or during the study span. Anonymously, the five general hospitals included in the study were given the codes from *H1* to *H5*. Drawing on the annual reports of the MOH (17,18), *H1* is a large and non-teaching hospital, *H2* (central and teaching complex), *H3* (large and non-teaching), *H4* (central and teaching complex) and *H5* (central and large teaching hospital). It is noteworthy that all the included hospitals deliver a similar range of health services, which in turn reduces the case mix bias and renders findings more comparable.

**Sampling and sample size**: All nurses, who have been working in the assigned departments at the hospitals under study, have experience for one year or more, and those who were willing to participate, have been included in the current study. Accordingly, the total number of those nurses in the five hospitals was 370. Of them, 70 nurses have been recruited for the pilot study to pretest the questionnaire used for data collection. Nurses whose total experience was less than one year, or were working in intensive care units, cardiology care units, and obstetrics departments, were excluded.

**Data collection**: By modifying the WHO responsiveness questionnaire, the questionnaire for this study was developed to fit the Palestinian context. The English version has been translated into Arabic by two professional persons, then back-translated into English once again by another two professionals. Content validity was checked by 12 experts from various health-related backgrounds. A pilot study encompassed 70 nurses has been carried out to examine the reliability of the used questionnaire which comprises two parts; 1) the nurses' sociodemographic characteristics including: age, sex, marital status, educational level, monthly income, position at work, overall experience, and experience in the current ward, in addition to the hospital of admission and medical department; and 2) the items related to inpatient care responsiveness. Totally, the questionnaire consists of 47 items; 10 were related to participants' characteristics, 36 domains' items on a 4-point Likert scale (1=highly disagree, 2=disagree, 3=agree, and 4=highly agree): DIG (5 items), CC (5 items), AUT (4 items), CON (3 items), PA (4 items), ASN (4 items), CP (3 items), QBA (8 items), and one more question on the importance of domains on a (0–10) scale. Drawing on the standardized items, Cronbach's alpha was applied to measure internal consistency of the aforementioned eight domains, and was as follow; 0.80, 0.76, 0.78, 0.79, 0.73, 0.75, 0.76, and 0.77, respectively. Data have been collected using the pretested interview-based questionnaire in the period between October 2019 and June 2020. Of the 300, 277 nurses have completed the questionnaire, thereby, the response rate was about 92.3%.

**Data analysis**: Data were collected by 10 well-trained health-care professionals (almost pharmacists) who are currently working in the study setting, and also familiar with the topic under study. Next, data were analyzed following assessment of data normality by the two-sample Kolmogorov-Smirnov test. Descriptive statistics (frequency, percentage, mean, and standard deviation), independent *t*-test and analysis of variance (ANOVA) were employed using SPSS 22.0 (SPSS Inc., Chicago, IL, USA). A Scheffé post-hoc test was also used to compare groups' means after ANOVA. For all tests, results were considered statistically significant at *p* value ≤ 0.05. Depending on the average scores, the weighted mean scores for domains were calculated based on the methodology recommended by the WHO (8). The percentage scores (%) used in Tamimi study were adopted as evaluation references as follow; (20–36% very low), (36.1–52% low), (52.1–68% moderate), (68.1–84% above moderate) and (84.1–100% high) (19).

**Ethical considerations**: The ethical approval for this study was granted by both the Vice-Chancellor in Research Affairs- Tehran University of Medical Sciences (IR. TUMS. VCR. REC. 1398.360), and the Helsinki committee in the Palestinian Health Research Council (PHRC/HC/959/19). Furthermore, informed consents were obtained verbally from all participants, and confidentiality was assured as well.

## Results

**Participants' characteristics**: As shown in Table 1, 277 respondents completed the questionnaire. The average age for participants was 33.09 years (SD = 7.713); 50.9% were female; 82.7% were married; 72.9% were holders of bachelor degree; 70.4% were staff nurses; 71.5% whose monthly income was between 900 and 1500 New Shekel (NIS) (1 USD = 3.39 NIS at the time of writing this manuscript); 52.7% were working in the surgical wards; the overall experience for 67.9% of the participating nurses was (-10) years; and 65.3% had experienced in the current ward for 1 to 5 years. Of the nurses, 17.4% have been working in hospital *H1*, 32.5% in *H2,* 14.8% in *H3,* 19.1% in *H4 and* 16.2% in *H5*.

**Table 1:** Socio-demographic characteristics of study participants

Variable		Number n – Percentage (%)
	H1	48 (17.4)
	H2	90 (32.5)
**Hospital**	H3	41 (14.8)
	H4	53 (19.1)
	H5	45 (16.2)
	21 – 25	40 (14.4)
	26 – 30	88 (31.8)
**Age**	31 – 35	59 (21.3)
**(years)**	36 – 40	43 (15.5)
	41 – 45	23 (8.3)
	46 – 60	24 (8.7)
	Mean = 33.097 SD = 7.713 years	
**Sex**	Male	136 (49.1)
	Female	141 (50.9)
	Single	40 (14.4)
**Marital status**	Married	229 (82.7)
	Divorced	8 (2.90)
	Diploma	50 (18.1)
**Educational level**	Bachelor	202 (72.9)
	High degree in nursing	17 (6.1)
	High degree (others)	8 (2.9)
	Practical nurse	53 (19.1)
**Position at work**	Staff nurse	195 (70.4)
	Head of division	12 (4.3)
	Head of ward	17 (6.1)
	900 – 1200 NIS	100 (36.1)
**Monthly income**	1201 – 1500 NIS	98 (35.4)
**(New Israeli Shekel -NIS)**	1501 – 2000 NIS	43 (15.5)
	≥2001 NIS	36 (13.0)
	Mean = 1550.25 SD = 566.65 NIS	
**Department**	Medical	131 (47.3)
	Surgical	146 (52.7)
	1 – 5 years	87 (31.4)
**Overall experience**	6 – 10 years	101 (36.5)
**(years)**	11 – 15 years	55 (19.9)
	16 years and more	34 (12.3)
	Mean = 9.039 SD = 5.876 years	
**Experience in the current ward**	1 – 5 years	181 (65.3)
**(years)**	6 – 10 years	68 (24.5)
	11 years and more	28 (10.1)
	Mean = 5.285 SD = 4.269 years	

**Scores of the responsiveness domains**: Based on nurses' views, the overall responsiveness in this study was “above moderate”, 78.06% and 76.93%, in both medical and surgical departments, respectively. ASN was the highest-performing domain, 95.5% and 93.1%, in the abovementioned departments, while, it was among the less important domains (Table 2). DIG was the second highest in performance, and it was followed by CON, CC and AUT in both departments. Regarding importance, DIG, CON, CC, and AUT were the 1^st^, 3^rd^, 5^th^, 2^nd^ in medical departments, whereas, they were the 1^st^, 2^nd^, 3^rd^, 5^th^ in surgical departments. For both departments, nurses have evaluated CP and QBA as the lowest domains in performance, and in importance as well (Table 2).

**Table 2:** Responses of participants (nurses) on responsiveness domains

#	Domain		Medical department					Surgical department				
			
		Wt. (WHO)	Mean (out of 4)	SD	Wt. score	Wt. P. (%)	Importance	Mean (out of 4)	SD	Wt. score	Wt. P. (%)	Importance
1	Dignity	12.5	3.601	0.569	11.25	90	1	3.438	0.377	10.74	85.92	1
2	Clear communication	12.5	3.357	0.303	10.49	83.92	5	3.272	0.414	10.23	81.84	3
3	Autonomy	12.5	3.280	0.366	10.25	82	2	3.205	0.446	10	80	5
4	Confidentiality	12.5	3.376	0.373	10.55	84.40	3	3.331	0.448	10.41	83.28	2

5	Prompt attention	20	2.822	0.390	14.11	70.55	4	2.791	0.547	13.95	69.75	4
6	Access to social support networks	10	3.820	0.337	9.55	95.50	6	3.726	0.442	9.31	93.10	7
7	Choice of care provider	5	2.315	0.528	2.89	57.80	7	2.292	0.643	2.87	57.40	8
8	Quality of basic amenities	15	2.392	0.365	8.97	59.80	8	2.512	0.450	9.42	62.80	6
	Overall responsiveness	100			78.06					76.93		

Wt. (WHO): weight of domains according to the WHO methodology; Wt. score = (Mean/4) × Wt. (WHO); Wt. P.: Weight percentage = [Wt. score/Wt. (WHO)] × 100 (%)

**Factors affecting responsiveness**: In the present study, age, sex and the overall experience of nurses have not resulted in statistically significant differences in the levels of responsiveness nor its domains. On the other hand, Table 3 shows that hospital of admission, marital status, educational level, position at work, income, hospital department, and the experience in the current ward have led to statistically significant differences in the level of overall responsiveness as well as some domains as reported by the participating nurses. By applying Scheffé test, remarkable differences were observed among the entire groups within the investigated variables as illustrated in (Table 4).

**Table 3:** Effect of study variables on responsiveness and its domains

Variable	Responsiveness domains	F	Significance (p ≤0.05)
**Hospital of admission**	All domains except confidentiality and prompt attention	-	≤0.05
**Marital status**	Access to social support networks	3.512	0.031
	Quality of basic amenities	8.119	0.000
	Dignity	3.468	0.017
**Educational level**	Autonomy	3.580	0.014
	Prompt attention	5.671	0.001
	Dignity	3.558	0.015
**Position at work**	Prompt attention	6.531	0.000
	Quality of basic amenities	3.796	0.011
**Monthly income**	Quality of basic amenities	2.725	0.045
	Dignity	2.836	0.005
	Access to social support networks	2.012	0.045
**Department**	Quality of basic amenities	-2.439	0.015
	Overall responsiveness	2.455	0.015
**Experience in the ward**	Autonomy	6.143	0.002

**Table 4:** Scheffé test for differences in responsiveness related to study variables (entire groups)

Domain	Variable (entire groups)	F	Significance (p ≤0.05)
**Dignity**	H1 –H3	0.318	0.046
	H1 –H3	0.339	0.000
**Clear communication**	H1 –H4	0.211	0.050
	H1 –H5	0.255	0.013
**Autonomy**	H1 –H3	0.268	0.037
	H1 –H5	0.281	0.019
**Access to social support**	H1 –H3	0.347	0.000
	H1 –H4	0.504	0.000
	H1 – H5	0.500	0.000
**Choice of provider**	H5 –H2	0.768	0.000
**Quality of basic amenities**	H4 –H1	0.536	0.000
	H4 –H2	0.535	0.000
	H4 –H5	0.242	0.024
**Access to social support**	Single – Married	-0.166	0.050
**Quality of basic amenities**	Single – Married	0.271	0.001
**Dignity**	PG studies (nursing) – PG studies (others)	0.585	0.045
**Autonomy**	PG studies (nursing) – Diploma	0.345	0.029
**Prompt attention**	PG studies (nursing) – Diploma	-0.329	0.101
	Head of division – Practical nurse	0.448	0.037
**Dignity**	Head of division – Staff nurse	0.450	0.019
	Head of division – Head of ward	0.514	0.045
**Prompt attention**	Practical nurse – Staff nurse	0.291	0.001
**Dignity**	Medical - Surgical	2.836	0.005
**Access to social support**	Medical - Surgical	2.012	0.045
**Quality of basic amenities**	Medical - Surgical	-2.439	0.015
**Overall responsiveness**	Medical - Surgical	2.455	0.015
**Autonomy**	≥11 years – (1–5) years	-0.287	0.002
	≥11 years – (6–10) years	-0.251	0.023

*NIS: New Israeli Shekel; PG: Postgraduate; BSc: Bachelor

## Discussion

This study aimed to assess the inpatient care responsiveness at the public general hospitals in the GS based on nurses' perspectives, and to determine the most influential factors on responsiveness level as well as its domains' performance. Commonly, assessment of performance and importance of domains are considered when investigating health system responsiveness (1,20).

In the present study, the overall responsiveness level was above moderate, 78.06% and 76.93%, according to nurses' perspectives in medical and surgical departments, respectively. This result seems satisfactory when check in the current status of the Palestinian health sector that suffers from scarce resources, shortage in workforces, and financial challenges due to multiple factors such as the long-term siege, imposed sanctions and internal political division. These findings were consistent with a Chinese study demonstrated that nurses' perspectives were higher than those of patients regarding quality of nursing care, and this can be attributed to the difference in their own views and expectations (21). In another study conducted by Javadi and colleagues, they aimed to compare between the perspectives of patients and nurses on responsiveness in the public and private hospitals in Isfahan, Iran (15). They found that the total responsiveness performance in both types of hospitals was similar (57.5%) based on nurses' opinions. In a third study, Turkish hospital managers rated their health system responsiveness 57.6% (22). This difference in responsiveness levels in both studies can be due to the lower expectation levels of the Gazan nurses when compared to their Iranian jobmates. The nurses in Gaza ought to consider the available resources and working under pressure, in addition to the limited capacity of the Palestinian health system to respond to the nonclinical needs of patients. Another reason can be added here that is awareness level about the concept of responsiveness among nurses in both countries, hence, educational and training activities are essential to improve the level of responsiveness (10). However, this was inconsistent with a previous study conducted in Italy showed that the increase in nurses workload has negatively affected the patients' perspectives on responsiveness (10).

Given the performance level of domains, it should be taken into account that the Palestinian hospital system is highly responsive to the interpersonal set more than the client-orientation domains, save ASN which was highest rated. DIG, CON, CC and AUT were rated 2^nd^, 3^rd^, 4^th^ and 5^th^ highest, respectively, in both departments. The Palestinian culture plays a paramount role in raising the level of ASN, as the family, relatives, friends and neighbors concern with supporting the patient in all aspects, moreover, the Palestinian health system is utterly responsive to social support networks, nonetheless, the formal social services are not provided by specialists in the MOH. Although DIG's level and importance varied from a country to another in the cross-country comparative study (23), its position in this study was similar to that in Javadi et al study as it was the second highest, and this reflects the attention paid to patients' respect during hospitalization in both countries. CON was the third highest (average 83.84%), but this was inconsistent with both Javadi et al, and Ugurluoglu and Celik studies, in which CON was rated first in both, 60.25% and 72.7%, respectively. However, the level of CON in this study indicates that the nurses believe that the Palestinian hospital system meets the expectation of inpatients regarding privacy issues to a satisfactory extent. Similar to Javadi et al findings, CP and QBA were the poorest-performing domains in the present study. This result for CP can be owned to the restricted policy of the Palestinian MOH which does not allow the patients to choose their providers due to the shortage in both specialists and well-qualified medical staff who can respond to patients' needs fairly in all hospitals. In short, the findings related to client-orientation domains showed that there is a room for further improvements that need special emphasis.

Generally, the interpersonal-related domains were ranked most important. DIG was the most important as stated by nurses in both departments. This result is anticipated for respect which is one of the main human rights that cannot be ignored when communicating inpatients regardless of their socioeconomic status. Despite ASN was the best-performing domain, it was ranked less important, which in turn delineates the preferences of people and how they prioritize their needs and expectations. CON was the third important in accordance to nurses' perspectives in medical departments, but the second in surgical ones. This indicates the importance of preserving the information private during treatment process. CP and also QBA were among the least important domains. For CP, this result was in line with De Silva and Valentine study in which it was weighted the least important (5%), but this was inconsistent with QBA weight which was (15%).

Among the nurses' characteristics; age, sex and overall experience did not result in any significant differences in the levels of responsiveness nor its domains. For sex, this was consistent with Javadi et al, but inconsistent with Ugurluoglu and Celik study in which males reported higher CON levels than females did, and the PA was rated higher by hospital managers whose overall experience was 21 years and more. Hence, the diminished effect of nurses' overall experience in this study can be attributed to their burnout, work overload, and cut of their salaries due to the governmental financial hardship and the Palestinian political conflicts. It is also worthy of mention that higher educational levels and positions of nurses were associated with better levels of DIG and AUT, but lower levels of PA. This can be justified in the light of their comprehensive view to the standards of care and health-care measures that should be fulfilled in this course, and this might also interpret the lower levels of AUT reported by the experienced nurses. In this regard, it is thought that these groups of participants used to appraise the current situation against the international standards, and reported their responses accordingly.

Hospitals *H1* and *H2* showed better levels of DIG and ASN, and CC and AUT, respectively, even though, these hospitals are large in size and have a wide range of casemix. This was consistent with Fiorentini et al study (10) in which they recognized a positive relationship between the casemix and the rise in all levels of responsiveness domains. Conversely, this was inconsistent with previous studies which demonstrated a negative association between the number of beds, and domains' levels and ultimately patients satisfaction (24,25). Furthermore, this also can be attributed to the size and qualification of medical and nursing staff that render them capable to meet patients' expectation, in addition to the good scale of economies that yielded from the effective coordination and cooperation in health-care teams. Considering the influence of clinical department, it is observed that the higher levels of overall responsiveness, DIG and ASN was an advantage for the medical departments which might reflect the awareness and focus of nursing staff on non-clinical aspects of health-care, while, the nurses at surgical departments reported better levels of QBA, which could be referred to the newly-established and well-furnished surgery buildings, and the package of services provided in more than one hospital.

Since most studies on responsiveness mainly focused on patients' experiences, supply-side should seriously be considered to delineate the *status quo* accurately. Despite of the satisfactory levels of interpersonal-related domains; DIG, CC, AUT and CON, there is a room for further improvements without extravagant expenditures. Policymakers in the Palestinian MOH need to pay more attention and allocate some extra budgets for the client-orientation domains, as the levels reported in the present study adversely affect the overall responsiveness of the Palestinian hospital system. The role of hospital characteristics and department was pivotal in creating significant differences in responsiveness level, hence, there is a need for robust researches to shed light on this black box. Additionally, some nurses' characteristics have substantially led to significant variations in their responses, accordingly, this calls for further mixed-method researches to get meaningful and realistic interpretation for the emerged findings. Differences of nurses' responses in both departments about the importance of domains draw the attention toward the socioeconomic status, cultural and values diversity among individuals, communities and even countries while measuring responsiveness. It is also recommended to carry out comprehensive studies in order to assess the responsiveness of the wide range of services; inpatient, outpatient, ambulatory … etc., which is provided to different groups of patients in the Palestinian society, public and private, and also considering the classification of nurses and other medical staff based on the employer and source of their salaries.
